# Transanal minimally invasive surgery (TAMIS) for local excision of benign and malignant rectal neoplasia: a 7-year experience

**DOI:** 10.1007/s00423-023-03217-4

**Published:** 2024-01-09

**Authors:** William P. Duggan, Niall Heagney, Sean Gray, Enda Hannan, John P. Burke

**Affiliations:** 1 Department of Colorectal Surgery, Beaumont Hospital, Dublin 9, Ireland; 2 Department of Physiology and Medical Physics, Royal College of Surgeons in Ireland, Dublin, Ireland

**Keywords:** TAMIS, Minimally invasive surgery, Rectal cancer, Surgical oncology

## Abstract

**Purpose:**

Transanal minimally invasive surgery (TAMIS) is an advanced transanal platform that can be utilised to perform high-quality local excision (LE) of rectal neoplasia. This study describes clinical and midterm oncological outcomes from a single unit’s 7-year experience with TAMIS.

**Methods:**

Consecutive patients who underwent TAMIS LE at our institution between January 1st, 2016, and December 31st, 2022, were identified from a prospectively maintained database. Indication for TAMIS LE was benign lesions not amenable to endoscopic excision or histologically favourable early rectal cancers. The primary endpoints were resection quality, disease recurrence and peri-operative outcomes. The Kaplan–Meier survival analyses were used to describe disease-free survival (DFS) for patients with rectal adenocarcinoma that did not receive immediate salvage proctectomy.

**Results:**

There were 168 elective TAMIS LE procedures performed for 102 benign and 66 malignant lesions. Overall, a 95.2% negative margin rate was observed, and 96.4% of lesions were submitted without fragmentation. Post-operative morbidity was recorded in 8.3% of patients, with post-operative haemorrhage, being the most common complication encountered. The mean follow-up was 17 months (SD 15). Local recurrence occurred in 1.6%, and distant organ metastasis was noted in 1.6% of patients.

**Conclusions:**

For carefully selected patients, TAMIS for local excision of early rectal neoplasia is a valid option with low morbidity that maintains the advantages of organ preservation.

## Introduction

Proctectomy with total mesorectal excision (TME) remains the standard of care for management of rectal cancer [1, 2]. Despite providing effective local tumour control, proctectomy with TME is associated with a significant risk of peri-operative morbidity, and a 3% risk of mortality [3]. From a quality of life (QOL) perspective, a significant proportion of patients will also require a temporary or permanent stoma, and for patients who undergo a low anterior resection, the incidence of low anterior resection syndrome is up to 45% [4, 5].

The widespread implementation of national screening programmes has led to a significant increase in rates of detection of early rectal neoplasms [6, 7]. This, coupled with advances in the scope and efficacy of modern neoadjuvant therapies, has resulted in a significant increase in the proportion of rectal cancers potentially amenable to local excision [8]. Consequently, there has been a growing trend towards implementation of less invasive procedures and rectum-preserving techniques.

There are two predominant rectum sparing approaches to transanal excision: transanal endoscopic microsurgery (TEM) and transanal minimally invasive surgery (TAMIS). Gerhard Buess first described TEM in 1984. It involves the use of a set of highly specialised instruments applied through a fixed metallic platform placed in the anal canal [9]. Atallah et al. first described transanal minimally invasive surgery (TAMIS) in 2010 [10]. TAMIS allows the use of standard laparoscopic cameras and instruments used in collaboration with a single-use flexible transanal access platform [11]. Some benefits of TAMIS over TEM include a lower associated cost of implementation, easier setup and greater procedural flexibility [12]. It is also assumed to have a more shallow learning curve due to the use of familiar laparoscopic cameras and instruments [11, 12]. At present, TAMIS is only recommended with curative intent for patients with pre-cancerous polyps; T1 rectal cancers; or small T2 rectal cancers, usually in the setting of clinical trials [13].

Current available evidence suggests local excision confers a QOL benefit over TME, whilst from an oncological perspective we await outcomes from ongoing randomised trials including the STAR-TREC and TESAR trials [14–18]. At present, much of the evidence pertaining to local excision in this context relates to TEM. Our unit, a tertiary referral rectal cancer centre, was an early adopter of TAMIS local excision in 2016. The objectives of this study are to present the clinical and midterm oncological outcomes for 168 consecutive patients who have undergone TAMIS LE of benign or malignant neoplasia at our institution, since implementation of the TAMIS LE programme.

## Methods

### Study population

The study population included a consecutive series of patients who underwent TAMIS between January 1st, 2016, and December 31st, 2022, as identified from a prospectively maintained institutional registry. Indications for TAMIS included (i) benign neoplasia not amenable to endoscopic excision, (ii) low-grade neuroendocrine tumours and (iii) stage I rectal adenocarcinomas with favourable histology (node-negative cT1, cT2 ≤ 3 cm in diameter, well differentiated, with absence of lymphovascular invasion). A pre-operative pelvic MRI was undertaken to determine depth of mural invasion and assess lymph node status in patients with either a confirmed pre-operative diagnosis of malignancy, concern for malignancy, or in instances where there was a question regarding suitability for LE. A computed tomography (CT) scan of the chest, abdomen, and pelvis and baseline carcinoembryonic antigen (CEA) completed the staging evaluation. Location of the lesion was assessed by digital rectal exam and rigid proctoscopy. Patients were discussed at a rectal cancer multi-disciplinary team (MDT) meeting prior to surgery. Patients with a cT2N0 tumour deemed unfit for a proctectomy were offered short-course radiotherapy in advance of TAMIS following MDT discussion (5 × 5 Gy) as per the TREC study [19]. Only patients who underwent TAMIS as a means of definitive local treatment of early rectal cancer were included in this study.

### Surgical technique

Patients underwent either full mechanical bowel preparation (for lesions in the upper rectum > 10 cm from anal verge) or received a phosphate enema prior to surgery. Patients were administered systemic antibiotics ≤ 30 min before incision according to hospital guidelines. All TAMIS procedures were performed by the same primary surgeon (JB), using the GelPOINT Path Transanal Access Platform (Applied Medical, Inc., Rancho Santa Margarita, CA). A high-definition 30° 10-mm camera was used in combination with standard laparoscopic graspers and a monopolar cautery device. Pnuemorectum was maintained with CO_2_ insufflation with flow set to 40 L/ min and pressure set to 15 mmHg. Where feasible, defect closure was performed using a V-Loc suture (Covidien-Medtronic, Minneapolis, MN).

### Follow-up

Standard post-operative clinical review was performed at 6 weeks. Flexible sigmoidoscopy was performed at 3 months to evaluate the scar. Patients with malignant neoplasms were followed according to National Comprehensive Cancer Network (NCCN) guidelines with a history, physical examination, MRI pelvis and CT thorax abdomen and pelvis and serum CEA level every 6 months for 2 years, then annually for a total of 5 years. A full colonoscopy was performed at 1 and 3 years after resection and every 5 years, thereafter, to identify metachronous lesions. For patients with excised specimens that revealed more advanced disease or histologically unfavourable features, discussion at a MDT meeting ensued. The alternative of a completion proctectomy in the setting of an invasive tumour diagnosis was discussed in all instances.

### Data collection

The primary endpoints were excision quality, clinical and oncologic outcomes. The following data was collected for each patient: age, sex, procedure type, body mass index (BMI), American Society of Anesthesiologists (ASA) score, distance from lesion to anal verge, primary pathology, TNM stage, lesion size, lymphovascular invasion, extramural venous invasion, perineural invasion, margin status, date of local and distant recurrence where applicable, length of inpatient stay (LOS), peri and post-operative complications when encountered. MRIs were reported by a specialist gastrointestinal consultant radiologist. Final MRI reports were inspected against corresponding histopathology reports to determine the sensitivity, specificity, positive predictive value and negativity predictive value of pre-operative MRI in differentiating malignant cancers from benign lesions. The resection margin was considered positive if malignant or dysplastic cells were found less than or equal to 1 mm from the circumferential resection margin or excised lesion perimeter. Recurrence rates were cross-checked by searching the departmental radiology database for each patient’s most recent surveillance imaging. Time to recurrence was defined as the time from the date of surgery to the date the first radiological or histological diagnosis of recurrence was made. Mean comprehensive complication index scores were calculated for each cohort to compare complication rates.

### Statistical analysis

Statistical significance was determined using Student’s *t* test or one-way ANOVA for continuous variables and the chi-squared test for categorical variables. Differences were considered significant if *P* < 0.05. The Kaplan–Meier survival analyses were used to describe the disease-free survival and a log rank test was performed with a *p* < 0.05 being considered statistically significant. All statistical analyses were performed using STATA 12.1 (StataCorp, College Station, TX).

## Results

### Clinical and pathological characteristics

There were 168 elective TAMIS LE procedures performed during the 7-year study period. Final surgical pathology revealed 102 benign and 66 malignant lesions (Table 1). Patients with malignant disease tended to be older (71 ± 11 V 66 ± 13 years,* P* = 0.043) and were more likely to have a higher ASA grade (*P* = 0.004). A pre-operative MRI was performed in 87 patients where there was a concern regarding depth of mural invasion (Table 2). A pre-TAMIS polypectomy was performed in 15/102 of the benign lesions and 18/66 of the cases of malignant disease (14.7% Vs 27.3%, *P* = 0.045). Benign lesions also tended to be larger in diameter than malignant lesions (52 ± 23 mm Vs 41 ± 22 mm, *P* = 0.005). There was no significant difference between the two groups in terms of sex, BMI or distance from lesion to anal verge.

**Table 1:** Patient, clinical and operative characteristics. Continuous variables are mean ± standard deviation, categorical variables are *n* (%)

	All (*n* = 168)	Benign (*n* = 102)	Malignant (*n* = 66)	*P* value
Age	68 ± 13	66 ± 13	71 ± 11	0.043
Male sex	101 (60.1%)	59 (57.8%)	42 (63.6%)	0.454
BMI, kg/m^2^	27 ± 3	27 ± 3	27 ± 3	0.488
ASA				0.004
I	27 (16.1%)	23 (22.5%)	4 (6.1%)	
II	78 (46.4%)	50 (49.0%)	28 (42.4%)	
III	62 (36.9%)	29 (28.4%)	33 (50.0%)	
Pre-TAMIS polypectomy	33 (19.6%)	15 (14.7%)	18 (27.3%)	0.045
Size (mm)	48 ± 23	52 ± 23	41 ± 22	0.005
Distance to anal verge (cm)	6 ± 4	6 ± 4	6 ± 4	0.752
Final pathology				
Benign	102 (60.7%)			
Adenoma* n*	100 (59.5%)			
Other *n*	2 (1.2%)			
Malignant	66 (39.3%)			
Adenocarcinoma* n*	59 (35.1%)			
ypT0	4 (2.4%)			
ypT1	3 (1.8%)			
ypT2	1 (0.6%)			
pT1	38 (22.6%)			
pT2	13 (7.7%)			
Carcinoid	6 (3.6%)			
Maltoma	1 (0.6%)			
Negative margin (R0)	160 (95.2%)	97 (95.1%)	63 (95.5%)	0.916
Resection depth				0.496
Full thickness	133 (79.2%)	79 (77.5%)	54 (81.8%)	
Submucosal	35 (20.8%)	23 (22.5%)	12 (18.2%)	
Tumour fragmentation	6 (3.6%)	4 (3.9%)	2 (3.1%)	0.775
Defect closure	161 (95.8%)	97 (95.1%)	64 (97.0%)	0.553

**Table 2:** Sensitivity, specificity, positive predictive value (PPV), negative predictive value (NPV) and accuracy of MRI in differentiating benign and malignant lesions on pre-operative MRI

	Sensitivity	Specificity	PPV	NPV
MRI (*N* = 87)	86.7%	19.1%	40.6%	69.2%

**Fig. 1:**
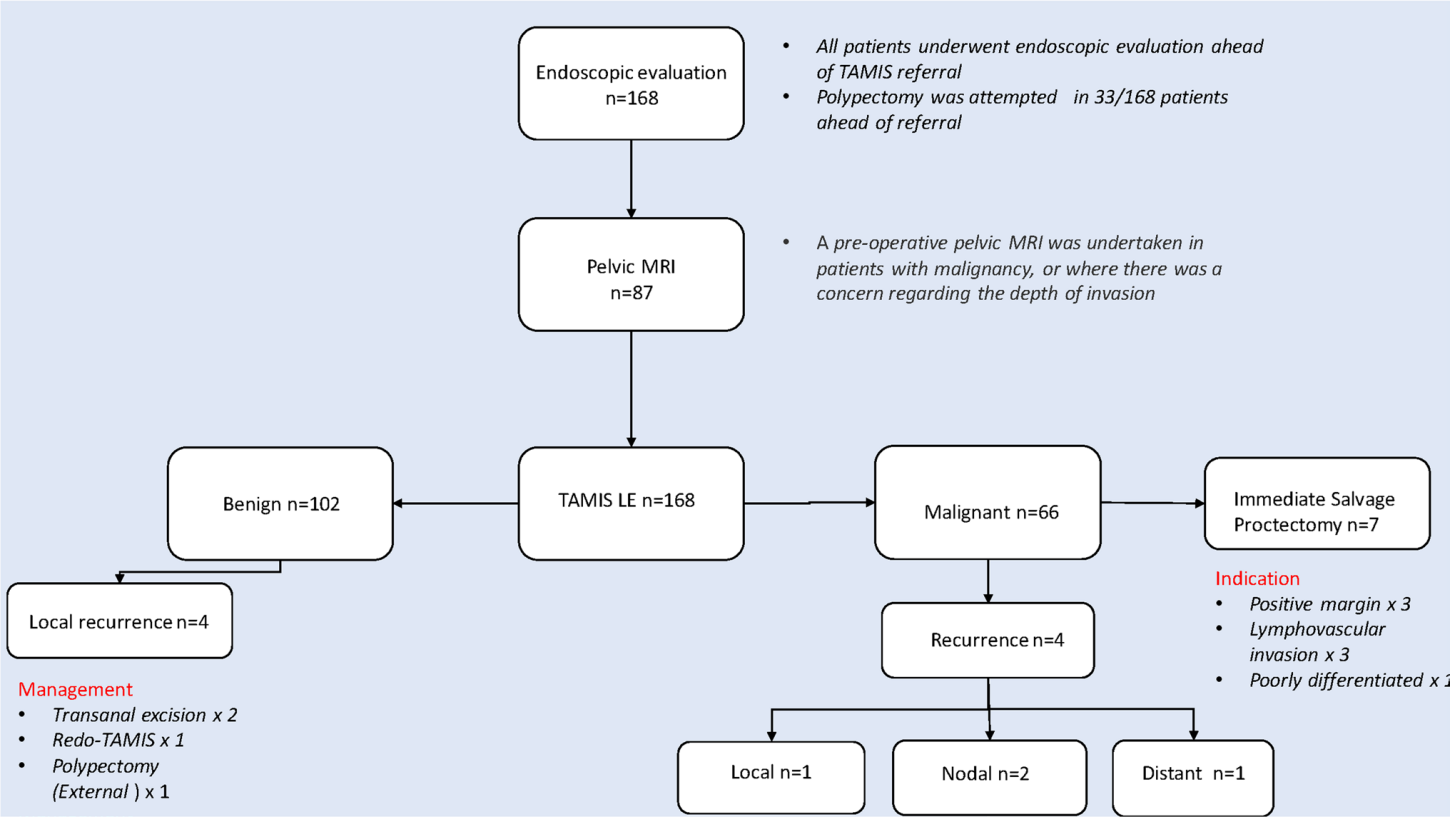
Retrospective algorithm briefly outlining firstly the pre-operative work up of patients who proceeded to TAMIS LE. We also outline the incidence of local recurrence and high-risk pathological features on primary resected TAMIS specimens. In each instance, the management strategy is detailed

13.6% of patients with adenocarcinoma (8/59) received neoadjuvant radiotherapy prior to surgery. 79.2% of excisions were full thickness (133/168), whilst 95.8% of defects were closed primarily (161/168). Tumour fragmentation was noted in 3.6% of cases (6/168). 95.2% of patients (160/168) received an R0 resection and there was no significant difference between the benign and malignant groups. With respect to the 8 patients with positive resection margins, of the 3 patients with malignant disease, all 3 proceeded to immediate salvage proctectomy. A further 4 patients with malignant disease who had negative margins also underwent immediate salvage proctectomy due to the presence of high-risk pathological features (Fig. 1). Of the 5 patients with positive margins and benign histology, 2 patients required re-excision due to regrowth. One patient underwent a Parks transanal excision, whilst the second patient underwent a re-do TAMIS. The remaining 3 patients did not recur.

### Peri-operative outcomes and length of inpatient stay

Peritoneal entry occurred in 4/168 cases (2.4%). All 4 lesions were benign and were located > 10 cm from the anal verge. All were re-approximated successfully via TAMIS. Post-operative complications were encountered in 8.3% of patients (14/168) and were twice as likely to occur in patients with malignant disease (12.1% vs 5.9%, *P* = 0.153) (Table 3). Post-operative haemorrhage was the most common complication encountered; all instances resolved without the need for intervention. The most significant complication occurred in a man who had received neoadjuvant radiation who returned on day 7 with perianal pain, bleeding and perianal discharge. A flexible sigmoidoscopy revealed a wound breakdown requiring endo-SPONGE (BRAUN, Konberg, Germany) treatment for 14 days. 89.9% of patients were successfully discharged on the day of or day 1 post-surgery.

**Table 3:** Peri-operative outcomes. Continuous variables are mean ± standard deviation, categorical variables are n (%). *CCI*, Comprehensive Complication Index

	All (n = 168)	Benign (n = 102)	Malignant (n = 66)	*P* value
Peritoneal entry Post-operative complications	4 (2.4%) 14 (8.3%)	4 (3.9%) 6 (5.9%)	0 (0.0%) 8 (12.1%)	0.105 0.153
Post-operative bleeding				
Required intervention	0	0	0	
No intervention	8 (Clavien I)	4	4	
Perianal pain	1 (Clavien I)	0	1	
Mild fecal incontinence	1 (Clavien I)	0	1	
Self-limiting fever	1 (Clavien I)	1	0	
Urinary retention	1 (Clavien I)	1	0	
Urinary tract infection	1 (Clavien II)	0	1	
Wound breakdown	1 (Clavien IIIb)	0	1	
CCI score		1 ± 2	2 ± 6	0.061
Length of stay	1 ± 4			0.143
LOS 0	83 (49.4%)	57 (55.9%)	26 (39.4%)	
LOS 1	68 (40.5%)	35 (34.3%)	33 (50.0%)	
LOS 2 +	17 (10.1%)	10 (9.8%)	7 (10.6%)	

### Oncological outcomes

The mean follow-up for patients who underwent TAMIS LE for malignant lesions was 20 months (SD 18) and 14 months (SD 11) for patients with benign disease (*P* = 0.008) (Table 4). Local recurrence occurred in 4.3% of patients with benign disease (4/92). All benign recurrences underwent further excision. Two patients with distal lesions underwent transanal excision; a further patient underwent a re-do TAMIS for a lesion at 9 cm. The final benign recurrence had an endoscopic polypectomy performed.

**Table 4:** Pathologic outcomes, peri-operative morbidity and long-term follow-up in patients who did not undergo immediate salvage radical surgery. Continuous variables are mean ± standard deviation, categorical variables are *n* (%)

	All (*n* = 155)	Benign (*n* = 92)	Malignant (*n* = 63)	*P* value
Mean duration of follow-up	17 ± 15	14 ± 11	20 ± 18	0.008
Local recurrence	5 (3.2%)	4 (4.3%)	1 (1.6%)	
Mean time to local recurrence	16 ± 8	18 ± 9	8	0.317
Nodal recurrence	2 (1.5%)	0 (0.0%)	2 (3.2%)	
Distant metastasis	1 (0.6%)	0 (0.0%)	1 (1.6%)	

**Fig. 2:**
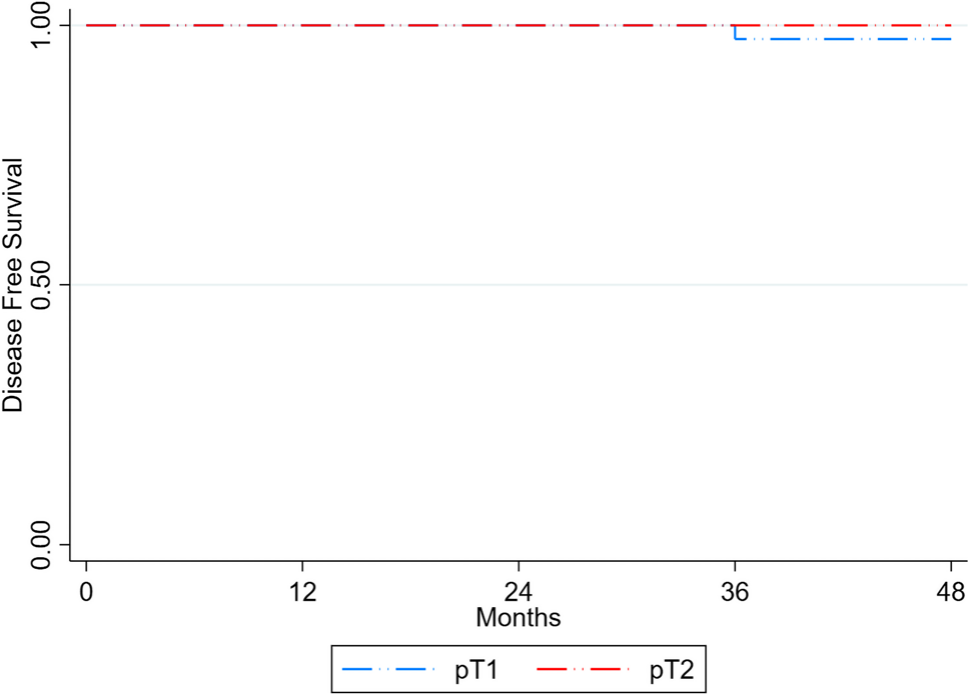
The Kaplan–Meier curves describing disease-free survival in 45 patients with invasive disease managed with TAMIS alone T1, *n* = 32, T2, *n* = 13. Excluded were patients who proceeded to salvage proctectomy and/or patients who received neoadjuvant chemoradiotherapy, *n* = 14

Regarding patients with malignant disease, mucosal recurrence occurred in 1 patient, nodal recurrence in 2 and distant organ metastasis in 1 (Table 4). The Kaplan–Meier disease-free survival curve for pT1 and pT2 rectal adenocarcinoma is shown in Fig. 2. The patient who developed local recurrence initially underwent an R0 resection for a pT1 tumour. Recurrence was identified on flexible sigmoidoscopy at 8 months; the patient was re-staged and received long-course radiotherapy followed by an anterior resection (Table 5); the tumour was definitively staged as ypT1N1. A further patient underwent a TAMIS LE of a 15-mm well-differentiated pT1 tumour. A surveillance pelvic MRI revealed a suspicious lateral pelvic sidewall node. This node was biopsied confirming the presence of adenocarcinoma. The patient received chemoradiotherapy, had a resolution of adenopathy and chosen for surveillance. At 38 months post TAMIS LE, the pelvic sidewall node was observed to regrow on MRI. The patient underwent an anterior resection with sidewall dissection, with the tumour definitively staged as ypT0N1. The second nodal recurrence occurred in a patient with initial pT2 histology, that demonstrated moderate differentiation and lymphovascular invasion. This patient was offered an immediate salvage proctectomy but declined. A suspicious mesorectal node was identified on pelvic MRI at 35 months. The patient underwent an endoscopic ultrasound and the node was biopsied confirming recurrence. The patient received adjuvant chemoradiation followed by an abdominperineal resection; the tumour was definitively staged as ypT0N1. A further patient received an R0 TAMIS LE of a pT1, moderately differentiated adenocarcinoma. Surveillance CT thorax, abdomen and pelvis at 36 months found a suspicious pulmonary nodule. This nodule was biopsied confirming the presence of colorectal adenocarcinoma. This patient was managed with systemic chemotherapy.

**Table 5:** Recurrences after TAMIS local excision of early rectal cancers

Patient	Original pathology	Size	Margin	Recurrence pathology	En bloc resection	Time to recurrence	Treatment of recurrence
1	pT1, moderately differentiated with LVI (patient declined further treatment)	35 mm	Neg	ypT1N1M0	Yes	8	CRT, followed by anterior resection
2	pT1, well differentiated	15 mm	Neg	ypT0N1M0	Yes	38	Anterior resection
3	pT2, moderately differentiated with	45 mm	Neg	ypT0N1M0	Yes	35	CRT, followed by APR
4	LVI (patient declined further treatment) pT1, moderately differentiated	50 mm	Neg	T0N0M1	Yes	36	Definitive chemotherapy

## Discussion

The implementation of a TAMIS LE programme has revolutionised the management of early rectal neoplasia in our institution. Many patients who previously were managed with TME are now offered a LE alternative in TAMIS which has been associated with acceptable peri-operative and oncological outcomes.

Previous studies have used measures such as tumour fragmentation and margin positivity rate to evaluate excision quality. 95.2% of our excisions were performed with negative margins and 96.4% were submitted without fragmentation. In a review of 200 consecutive TAMIS LE procedures, Lee et al. reported a negative resection margin rate of 93% whilst 95% of tumours were submitted without fragmentation [20]. Meanwhile, some smaller studies have reported contrasting results, Haugvik et al. found that 22% of patients had a positive resection margin following TAMIS LE of benign rectal lesions, whilst 31% of specimens in this study were too fragmented to allow reporting of marginal status [21]. Mohamed et al. report findings from a cohort of 42 patients who underwent TAMIS LE; the positive margin rate was 4% in this study; however, 19% of specimens again were submitted in fragments [22]. From a technical perspective, 79.2% of our patients underwent full thickness excision, compared to only 14% in the Mohamed et al. group [22]. The authors hypothesize that by carrying out full thickness excisions as standard, the likelihood of fragmenting the tumour is significantly reduced. It is also notable that in both our study and the study by Lee et al., surgical volume was significantly higher than in the other aforementioned studies [20–22]. In our single surgeon study, 168 TAMIS procedures were performed over 7 years, whilst in the findings reported by Mohamed et al., 42 excisions were performed by 2 surgeons over a 5 and a half–year period; similarly in the study by Haugvik et al., 51 procedures were performed over a 4-year study period [21, 22]. There is a likely association between surgical volume and excision quality in this context.

Our recurrence rate was 6.4% for malignant lesions, with only 1 instance of mucosal recurrence. Lee et al. reported a local recurrence rate of 6% for patients with malignant disease treated with TAMIS LE [20]. These oncological outcomes compare favourably with data obtained from TEM series. Moore et al. reported a local recurrence rate of 8% for malignant lesions, whilst Christoforidis et al. reported a 15% 5-year local recurrence rate after TEM [23, 24]. A critical juncture in the management of patients post TAMIS LE is how to proceed when initial primary histology reveals high-risk pathological features. In our single experience with mucosal recurrence, the patient was offered further treatment due to lymphovascular invasion on primary histology, but declined. Similarly in the series by Lee et al., 3 of 6 patients who developed recurrence declined further treatment initially despite the presence of high-risk pathological features. Four further patients in our cohort proceeded to immediate salvage proctectomy, due to the presence of lymphovascular invasion or poor differentiation on their initial pathology; all 4 had positive definitive outcomes, none requiring an unnecessary permanent ostomy. Apart from 1 case of mucosal recurrence, we also report 2 incidences of nodal recurrence and a singular instance of distant metastatic disease, diagnosed 3 years post TAMIS LE. None of these 3 patients was in receipt of scheduled neoadjuvant or adjuvant systemic therapy as part of their management, and a key question is whether it could have made a positive impact in this context? At present, there remains no definitive consensus on the use of systemic chemotherapy and radiotherapy in combination with local excision [25, 26]. Data from the TREC study suggests short-course radiotherapy followed by TEM may achieve high levels of organ preservation, with relatively low morbidity and indications of improved quality of life over radical resection; however, larger randomised studies, such as the ongoing STAR-TREC and TESAR trials, are needed to more precisely determine oncological outcomes following different organ preservation treatment schedules [17–19].

The overall post-operative complication rate across our TAMIS cohort was 8.3%. Lee et al. reported post-operative morbidity in 11% of patients [20]. A further large multi-centre study which included 428 patients compared outcomes post TEM and TAMIS, and found no significant difference in post-operative complication rate amongst the 2 methods of local excision (TEM 11% vs TAMIS 9% *P* = 0.477) [27]. The most common post-operative complication encountered across our cohort was post-operative haemorrhage. The most significant complication in our cohort occurred in a patient who received neoadjuvant radiotherapy. Similarly in the series by Lee et al., patients who received radiotherapy in advance of surgery were more likely to encounter wound-related complications or impaired post-operative functionality [20].

Selecting appropriate patients for local excision can be difficult and is limited significantly by the efficacy of radiological investigative techniques in determining pre-operative depth of local mural invasion. MRI was associated with a modest sensitivity of 86.7%, and a specificity of only 19.1% in this study. Similarly, a previous systematic review found endoscopic rectal ultrasound to be associated with a sensitivity of only 50% and a specificity of 89% in determining occult T1 rectal cancers pre-operatively [28]. These findings are important and suggest clinicians and multi-disciplinary forums should interpret pre-operative radiological investigations with caution before proceeding with LE.

The authors acknowledge that this study has important limitations. Most notably, the retrospective nature of data analysis can introduce selection bias that may affect the study’s veracity. This study is also limited because all TAMIS operations were performed by a single-experienced colorectal surgeon at a single high-volume institution, which may limit the generalizability of the results.

## Conclusions

For carefully selected patients, TAMIS LE of rectal neoplasia is a valid option with low morbidity and comparable midterm oncological outcomes.

## Data Availability

Original data may be made available upon reasonable request.
